# Sex Differences in Force, Velocity, and Power Percent Changes During Countermovement Jump Performance Following a Dynamic Warm-Up

**DOI:** 10.3390/muscles5010004

**Published:** 2026-01-09

**Authors:** Gabriel J. Sanders, Maura Bennett, Roger O. Kollock, Corey A. Peacock

**Affiliations:** 1 College of Education, Criminal Justice, and Human Services, University of Cincinnati, Cincinnati, OH 45221, USA; 2 College of Allied Health Sciences, University of Cincinnati, Cincinnati, OH 45221, USA; 3 Health & Natural Sciences: Kinesiology & Rehabilitative Sciences, University of Tulsa, Tulsa, OK 74104, USA; 4 College of Healthcare Sciences, Nova Southeastern University, Fort Lauderdale, FL 33328, USA

**Keywords:** neuromuscular, performance, stretch-shortening

## Abstract

**Background**: The study examined sex differences in countermovement jump (CMJ) force plate metrics and neuromuscular responses to a standardized dynamic warm-up in physically active college students. **Methods**: Forty-one participants (21 males, 20 females) completed pre- and post-warm-up assessments of CMJ performance using a dual force plate system. Body composition was measured via bioelectrical impedance analysis, and performance metrics included force, velocity, power, and other jump metrics. Percent change scores were calculated for all metrics. **Results**: Males demonstrated significantly greater improvements in braking force metrics compared to females, including force at minimum displacement (11.4% Δ male vs. 5.7% Δ female, *p* = 0.043), average braking force (10.6% Δ male vs. 5.0% Δ female, *p* = 0.043), and peak braking force (11.5% Δ male vs. 5.7% Δ female, *p* = 0.043). No significant sex differences were found in velocity, power, propulsive force, or other general CMJ performance variables. Hierarchical regression analyses revealed that sex was a significant (*p* ≤ 0.043 for all) predictor of changes in braking force metrics, while lean body mass did not enhance model fit or independently predict force changes. The addition of lean body mass slightly attenuated the sex effect but did not contribute meaningfully to the models. **Conclusions**: Findings suggest males may experience greater braking force adaptation to a dynamic warm-up, while other performance outcomes appear similar between sexes. These results may inform sex-specific warm-up strategies targeting neuromuscular readiness and braking force development.

## 1. Introduction

Explosive movements are essential in many sports, especially at competitive levels, where athletes must frequently perform high-intensity actions with minimal rest during both training and competition [1,2]. In team sports, one such movement, the countermovement jump (CMJ), is especially common in disciplines like basketball and volleyball, where it is considered a fundamental motor skill [3,4,5]. CMJs consist of an eccentric (braking) phase followed by a concentric (propulsive) phase and are often repeated under varying intensities with limited recovery [6]. While jump height is often used in sport contexts to evaluate performance, research more frequently employs metrics such as peak or average force, velocity, and propulsive impulse to assess jump quality [7,8].

To maximize performance in these tasks, athletes typically use warm-up routines. The general relationship between warm-ups and performance tends to be positive, though evidence suggests that the effectiveness of warm-ups is greatest when tailored to the individual [7]. These benefits are evident in tasks such as running, jumping, and cycling [9]. Improvements following a warm-up may be attributed to increases in muscle temperature, enhanced nerve conduction, muscle volume, and post-activation performance enhancement (PAPE) [9,10,11,12,13]. PAPE refers to a temporary increase in muscular force and power output following high-intensity conditioning activities such as plyometrics, isometrics, or explosive movements [14,15,16]. However, the presence and magnitude of PAPE depend on the intensity, type, and timing of the conditioning activity as well as individual factors such as sex, training experience, and relative strength.

Though PAPE has been widely studied in men, it is likely underexplored in women. Research to date shows mixed findings as one study reported both sexes experienced performance improvements up to three minutes post-conditioning, but women’s performance declined thereafter, suggesting a shorter window of benefit [12]. In contrast, another investigation found that female athletes displayed their greatest PAPE response approximately 12 min after the conditioning activity [17]. A third study observed no immediate improvement in elite female volleyball players but noted a delayed enhancement occurring six hours later [18]. Collectively, these differing results indicate that sex-specific PAPE responses are complex and may be shaped by differences in rest intervals, sport-specific demands, or underlying physiological characteristics of the individual.

Not all warm-up strategies produce equal results, particularly when considering variables such as sex, training status, and relative strength. For example, when male and female athletes were matched for relative strength, sex-based differences in PAPE were no longer significant [19]. The type of stretching included in a warm-up may also influence performance. Static stretching, defined as holding a stretch at maximal or submaximal intensity for 30–60 s, has been widely used by performance coaches in applied settings. However, dynamic stretching, which involves active movement through a large range of motion, is now generally considered more effective. Some studies report that static stretching does not impair performance in trained individuals relative to dynamic methods [9], while others find that dynamic stretching improves explosive movement performance such as jumping [10]. Additionally, plyometric warm-ups show promise in enhancing power output through PAPE-related mechanisms [11,14]. These results underscore the need for warm-up strategies to be customized to the athlete’s physiological and performance profile to optimize outcomes and avoid counterproductive routines.

Despite a growing body of research, findings on warm-up effects on CMJ performance remain inconsistent. This variability is likely due to differences in protocol design and participant characteristics. Some studies confirm that dynamic stretching is the most effective in maintaining or increasing jump height relative to static or no stretching [10,11]. Other research shows no significant differences between stretching types, though these studies are often conducted in trained populations, which may be more resistant to the negative effects of suboptimal warm-ups [8]. For instance, in trained males, static stretching appears to negate the benefits of a general warm-up on vertical jump performance [8], while dynamic stretching enhances lower-body explosiveness [8,20]. Further, plyometric-based warm-ups have been shown to positively affect jump performance in mixed populations that include both trained and untrained individuals [7].

Strength and muscle fiber composition differences between sexes may explain variations in CMJ performance and may also influence warm-up efficacy [21]. Differences in muscle stiffness and fatigue profiles could also cause men and women to respond differently to static or dynamic warm-ups [7]; For instance, when ballistic jumping was incorporated into the warm-up of middle-distance runners, males improved their 1000 m performance, likely due to enhanced anaerobic performance in the first 250 m [21]. In contrast, females experienced less benefit, potentially due to increased fatigue and slower lactate clearance [21]. While most research focuses on jump height as the primary outcome, few studies evaluate how warm-up protocols affect force production, velocity, or propulsive characteristics, especially in a sex-comparative context [22]. Given this gap, braking-phase metrics may offer additional insight because eccentric control precedes concentric force production and may be more sensitive to subtle warm-up–related changes in neuromuscular readiness than a general performance measure such as jump height.

Therefore, the purpose of this study was to investigate sex differences in force plate performance metrics, specifically average and peak CMJ measures, between males and females under two conditions: pre-warm-up and post-warm-up. Examining these differences is valuable because males and females may not respond to warm-ups in the same way, given well-established variations in muscle mass, fiber type distribution, tendon stiffness, and hormonal influences. A standardized dynamic warm-up is commonly prescribed to enhance neuromuscular readiness and optimize explosive performance, yet little is known about whether the performance benefits are similar across sexes. Understanding whether males and females exhibit different neuromuscular responses has practical implications for tailoring warm-up strategies to maximize performance outcomes or even reduce the likelihood of injury.

Additionally, this study explored whether sex-specific variations in body composition are associated with differential changes in CMJ performance. Since males typically possess greater absolute lean mass, they may exhibit higher peak force and jump height values, but when expressed relative to body composition (e.g., lean body mass), these sex-based differences may diminish. Identifying whether warm-up responses are more strongly tied to muscle mass or sex itself can provide insight into the mechanisms driving performance adaptations. It was hypothesized that males would demonstrate higher absolute neuromuscular performance metrics (e.g., peak force and jump height) in both pre- and post-warm-up conditions. However, when normalized for lean mass, no significant differences were expected between sexes. Finally, it was anticipated that the dynamic warm-up would elicit greater relative improvements in CMJ performance in males, with body composition variables showing stronger associations with performance changes in males compared to females.

## 2. Methods

### 2.1. Participants

Forty-one physically active college students (21 males: 20.7 ± 1.8 years; 20 females: 21.7 ± 2.5 years) were recruited for this investigation. The sample size of 41 participants was deemed sufficient based on similar sex-comparison studies examining acute neuromuscular responses to warm-up interventions, providing adequate statistical power to detect between-sex differences [7,10,11,23]. Descriptive data on participant physical characteristics are presented in Table 1. All assessments were conducted during a single visit to the university’s Human Performance Laboratory. Upon arrival, participants completed the Physical Activity Readiness Questionnaire (PAR-Q+) to screen for any contraindications to exercise. Eligibility required that participants reported engaging in structured exercise at least three days per week. Once eligibility was confirmed, the study protocol was thoroughly explained, and participants were encouraged to ask questions. Each participant then signed an informed consent form approved by the university’s Institutional Review Board (IRB) prior to participation. To control external factors that might influence neuromuscular performance outcomes, participants were advised to abstain from vigorous exercise and caffeine consumption for a minimum of 24 h before testing.

### 2.2. Procedure

Following informed consent, participant profiles were established in both the Hawkins Dynamics dual force plate system and the InBody 770 body composition analyzer. These profiles included identifiers such as the participant’s full name and the date of testing, which were used consistently throughout data collection to ensure accurate dataset alignment. Baseline and post-intervention assessments included handgrip strength (measured using a validated digital hand dynamometer; Camry EH101, Sensun Weighing Apparatus Group Ltd., Zhongshan City, China), body composition (InBody 770, Seoul, Republic of Korea), and CMJ performance. CMJ data were captured using a research-grade portable dual force plate (Hawkin Dynamics, Westbrook, ME, USA) operating at 1000 Hz, a system previously validated for measuring vertical jump metrics.

To support consistent data tracking, all measurements were cataloged by name and date for merging datasets at the conclusion of data collection. The assessment sequence was standardized, with handgrip strength evaluated first, followed by CMJ testing. For grip strength, two trials were completed with the dominant hand in a fully extended position; the highest value (kg) was recorded. Consistent with previous research [22,24], CMJ trials were performed with participants placing their hands on their hips to minimize arm contribution, completing two maximal effort jumps; both attempts were retained for analysis.

All testing sessions were conducted indoors under stable environmental conditions. Participants were instructed to wear athletic attire and supportive footwear to ensure safety and consistency. Before post-test assessments, participants engaged in a standardized 10-min dynamic warm-up intended to elevate neuromuscular readiness. This warm-up included plyometric elements typical of collegiate training and consisted of three progressive phases: dynamic preparation (Phase 1), movement preparation (Phase 2), and potentiation (Phase 3).

Warm-up movements were carried out over a standardized distance of 9.1 m (10 yards). During Phase 1, participants completed two jogging passes (totaling 18.2 m), followed by a sequence of dynamic locomotor drills including shuffling with arm swings, carioca with hip rotation, forward and backward skips, lateral skips, and power skips aimed at maximizing both height and distance. Phase 2 emphasized dynamic mobility, incorporating quad pulls with reach, hamstring scoops, knee hugs with calf raises, reverse hip openers, ankle pulls into side lunges, and forward lunges with a torso twist, all performed across 9.1 m. This phase also included three static quadricep and hamstring stretches per leg and five single-leg hip abductions. Phase 3 consisted of plyometric activities: forward and backward bilateral hops over 4.6 m in each direction, lateral bilateral hops, alternating hops in both forward/backward and lateral directions over 9.1 m and concluded with 10 vertical pogo jumps.

Following the completion of the warm-up routine, post-assessments for handgrip strength and CMJ tests were conducted using the same procedures as the initial testing session. Participants completed two trials of post-warm-up grip strength, followed by two CMJ attempts. After testing was concluded, all individual data files were securely organized and placed into the finalized participant folder to be included in the subsequent data analysis phase.

### 2.3. Statistical Analysis

Descriptive statistics (means ± SD) were calculated by sex for all variables, including age, height, weight, and body composition metrics. Percent change scores from pre to post assessment were calculated for all countermovement jump (CMJ) variables (force, velocity, and power) using the formula: ((Post − Pre)/Pre) × 100. Independent samples *t*-tests were conducted to examine sex differences in percent change for both peak and average CMJ metrics. This approach enhances the interpretability of performance changes by accounting for initial differences in baseline performance, which is important when comparing responses across sexes.

Assumption testing was performed for all relevant comparisons prior to inferential analyses. Normality was evaluated using the Shapiro–Wilk test, and homogeneity of variances was assessed using Levene’s test. Cohen’s d effect sizes were calculated for each independent samples *t*-test to evaluate the magnitude of sex differences in percent change scores, with effect sizes interpreted as small (d = 0.2), medium (d = 0.5), and large (d = 0.8), in accordance with Cohen’s guidelines. Confidence intervals for regression coefficients were also included to provide additional context for the precision of the estimated effects.

Pearson correlations were used to examine relationships between body composition variables and changes in CMJ force metrics. When stratified by sex, correlations were no longer statistically significant (females: *r* = 0.339, *p* ≥ 0.144 for all and males: *r* = 0.323, *p* > 0.153 for all), implying potential confounding or moderation by sex. Therefore, multiple linear regression models were conducted to determine whether sex, body composition, or their interaction predicted changes in force at minimum displacement, average braking force, and peak braking force. Interaction terms (e.g., sex × body composition) were included to test for potential moderation effects. All statistical analyses were conducted using IBM SPSS 29.0 (Version 29.0, IBM Inc., Armonk, NY, USA). The criterion for statistical significance was set a priori at *p* ≤ 0.05.

## 3. Results

### 3.1. Physical Characteristics 

Participant age, height, weight, percent body fat, lean body mass, and skeletal muscle mass are presented in Table 1. Assumption checks indicated that normality and homogeneity of variance criteria were met for all analyses.

### 3.2. Force-Based Metrics

Independent *t*-tests revealed males demonstrated greater percentage increases than females in force at minimum displacement, average braking force, and peak braking force presented in Table 2. No significant sex differences were found in the percentage change for average or peak propulsive force. These findings imply that males may experience greater neuromuscular adaptation in braking force characteristics in response to a standardized warm-up, whereas propulsive force responses appear similar between sexes.

### 3.3. Velocity-Based Metrics 

Independent *t*-tests revealed no significant sex differences in the percentage change for any velocity-based metrics, including average and peak braking velocity as well as average and peak propulsion velocity (Table 3). These results suggest that males and females exhibit similar velocity adaptations in both braking and propulsion phases of the countermovement jump following a standardized dynamic warm-up.

### 3.4. Power-Based Metrics 

Independent *t*-tests revealed no significant sex differences in the percentage change for power-based metrics, including average and peak braking power as well as average and peak propulsive power (Table 4). These findings indicate that males and females demonstrate comparable power adaptations in both braking and propulsion phases in response to the dynamic warm-up.

### 3.5. Grip and CMJ Metrics

Independent *t*-tests revealed no significant sex differences in the percentage change for grip strength, jump height, flight time, braking rate of force development (RFD), jump momentum, or CMJ depth (Table 5). It is possible both males and females experienced similar adaptations across general performance metrics and force-time characteristics of the countermovement jump following the dynamic warm-up.

### 3.6. Regression

Hierarchical regression analyses were conducted to examine whether sex and lean body mass predicted changes in countermovement jump (CMJ) braking force metrics following a standardized dynamic warm-up (Table 6). Three primary performance outcomes were evaluated: force at minimum displacement, average braking force, and peak braking force.

In each model, sex was entered at Step 1, followed by lean body mass at Step 2. For all three outcomes, the initial model including sex as the sole predictor was statistically significant. In this model, sex significantly predicted changes in force metrics, with males showing greater improvements in braking force responses than females.

When lean body mass was added in the second step, the models demonstrated a slight increase in explained variance; however, this increase was not statistically significant for any outcome. Furthermore, lean body mass did not emerge as a significant independent predictor in any of the Step 2 models. The beta coefficients for sex were attenuated in the second model, but remained non-significant, indicating that lean body mass did not meaningfully enhance the prediction of braking force adaptations beyond the effect of sex alone. Given these results, only the first model (sex-only) was retained for interpretation across all outcomes. The final regression models reveals that sex is a predictor of neuromuscular responsiveness to dynamic warm-up, whereas lean body mass contributes minimally to explaining individual differences in short-term braking force adaptations.

## 4. Discussion

The primary finding of this study is that males demonstrated greater acute improvements in braking-force metrics than females. Males demonstrated larger percent increases in force at minimum displacement (11.4% vs. 5.7%), average braking force (10.6% vs. 5.0%), and peak braking force (11.5% vs. 5.7%) compared to females, indicating a more pronounced response in eccentric braking performance. Regression analyses further identified sex as a significant independent predictor of braking force changes, whereas lean body mass did not improve model fit or add explanatory power. However, it is important to note that these findings may reflect a combination of factors beyond biological sex alone. Differences in baseline strength, age-related neuromuscular development and familiarity with jump-based tasks, or psychological factors such as task engagement or motivation may also have contributed to the observed responses [25,26]. In contrast, no significant sex differences were detected in velocity, power, or general CMJ outcomes (e.g., jump height, grip strength, flight time), suggesting that the warm-up protocol selectively influenced braking-related metrics in males, while other domains remained unaffected. These findings emphasize the value of assessing braking-specific force metrics and support the need for sex-specific considerations when designing warm-up strategies to optimize neuromuscular readiness.

The current study found that males exhibited greater acute improvements in braking force metrics than females following a dynamic warm-up, while no significant sex differences were observed in velocity, power, or general CMJ outcomes. These results align, in part, with previous findings that dynamic warm-ups and plyometric activities can enhance explosive performance, particularly in jumping tasks, through mechanisms such as post-activation potentiation and increased muscle temperature [9,10,11]. However, it is important to note that the present study did not directly assess underlying muscular mechanisms. While earlier studies focused largely on outcomes like jump height or general explosiveness, the present study adds new insight by highlighting braking force as a specific domain where sex differences emerge. Previous literature has consistently shown dynamic stretching to be more beneficial than static stretching for jump-based tasks, but often lacks detailed analysis of sex-specific adaptations across multiple force metrics [7,11].

In contrast to studies that report minimal differences in CMJ outcomes between stretching protocols in trained populations [8,9,10], our results reflect that braking force characteristics may be more sensitive to warm-up effects and more likely to reveal sex-based differences. While prior research states that trained individuals may be protected from the negative effects of static stretching [8,9,10], the participants in this study were not sport-specific athletes, which may explain the clearer separation in performance gains between males and females. Interestingly, although our results agree with previous findings that dynamic warm-ups benefit jump performance [10,11], the advantage in braking force observed among males could relate to underlying physiological factors such as fiber type composition, muscle stiffness, or neural recruitment efficiency, which were not directly assessed in this study but have been hypothesized to influence warm-up response [7,21].

The literature also implies that sex-specific adaptations to warm-up may stem from inherent biological differences, including muscle fiber composition, hormone profiles, and metabolic recovery patterns [21]. For instance, previous research reported that ballistic jumping as part of a warm-up improved middle-distance running performance in males but not females, with the proposed explanation being slower lactate clearance and lower potentiation in females [21]. This aligns with the current study’s finding that males responded more strongly to the warm-up in terms of braking force, suggesting a more favorable acute neuromuscular response. Furthermore, while earlier studies often relied on jump height alone as an outcome measure [27], the present study provides a more nuanced assessment by evaluating braking force, a key indicator of eccentric strength and readiness. These findings reinforce the value for sex-specific considerations in warm-up design, as males may derive greater benefit from high-intensity, plyometric strategies, particularly when targeting braking performance, possibly due to differences in muscle fiber composition, tendon stiffness, and hormonal influences that modulate neuromuscular potentiation [28,29].

Although this study provides new evidence on sex-specific responses to a dynamic warm-up, certain limitations must be acknowledged. First, the warm-up protocol used in this investigation included a high volume of plyometric movements, particularly during the final phase. This explosive, jump-focused design may have created conditions that accentuated male performance advantages, especially in braking force variables. As a result, the sex differences observed may not generalize to warm-ups with lower intensity, fewer plyometric elements, or differing movement demands. Second, participants were recreationally active, college-aged individuals without specialized training backgrounds, and therefore important factors such as training history, relative strength, and baseline performance variability could not be quantified or included as covariates in the regression models. Therefore, the applicability of these findings to elite athletes remains uncertain. Highly trained female athletes in sports that involve frequent jumping, such as volleyball, may show adaptation patterns more comparable to males, while athletes from lower-jump-demand sports may exhibit larger sex disparities in response to the same warm-up stimulus.

Lastly, the absence of neuromuscular or physiological markers beyond performance-based CMJ metrics. The study did not include electromyographic data, stiffness measures, or muscle activation profiles, which could have helped clarify the mechanisms driving the observed sex differences. Also, this study involved multiple comparisons, therefore *p*-values near 0.05 should be interpreted with caution. Future studies should incorporate such tools to gain a more complete understanding of how males and females respond at the muscular and neural levels to different warm-up strategies.

## 5. Conclusions

This study suggests that males may experience greater acute improvements in braking force during CMJ following a dynamic warm-up compared to females. These effects were not observed for velocity, power, or general CMJ metrics. Findings highlight braking force as a potentially sensitive indicator of warm-up responsiveness, though larger and more diverse samples are required to confirm sex-specific patterns. For practitioners, braking force should be monitored when evaluating readiness and warm-up effectiveness, as it may reveal subtle sex-based differences not captured by traditional jump height or power measures. Coaches and strength professionals working with recreationally trained or physically active college populations can use this information to individualize warm-up protocols, ensuring that both male and female athletes are optimally prepared for subsequent training or competition. 

## Figures and Tables

**Table 1:** Compares physical characteristics and body composition variables by sex.

	Female (*n* = 20)			Male (*n* = 21)		
Age (years)	21.8	±	3.6	20.7	±	1.8
Height (cm)	161.2	±	7.2	180.8	±	6.9 *
Weight (kg)	62.3	±	10.5	83.1	±	10.7 *
Percent Body Fat (%)	25.8	±	6.7	15.0	±	8.1 *
Lean Body Mass (kg)	45.8	±	6.2	70.3	±	9.0 *
Skeletal Muscle Mass (kg)	25.3	±	3.7	40.5	±	5.5 *

Data are Means ± SD. * Significant difference between males and females, *p* < 0.001.

**Table 2:** Demonstrates percent changes from pre to post warmup by sex for average and peak braking and propulsion force-based metrics.

Force-Based Metric	Female % Δ			Males % Δ			*t*-Statistic	*p*-Value	Effect Size Cohen’s *d*
Force at Minimum Displacement (N)	5.7	±	6.4	11.4	±	10.7	2.087	0.043	0.65
Average Braking Force (N)	5.0	±	5.2	10.6	±	8.5	2.547	0.015	0.80
Peak Braking Force (N)	5.7	±	6.5	11.5	±	10.7	2.088	0.043	0.65
Average Propulsive Force (N)	3.6	±	3.3	4.6	±	4.3	0.826	0.414	0.26
Peak Propulsive Force (N)	4.0	±	3.9	5.4	±	7.4	0.792	0.433	0.25

Data are Means ± SD.

**Table 3:** Shows percent changes from pre to post warmup by sex for average and peak braking and propulsion velocity-based metrics.

Velocity-Based Metric	Female % Δ			Males % Δ			*t*-Statistic	*p*-Value	Effect Size Cohen’s *d*
Average Braking Velocity (m/s)	12.2	±	13.5	14.5	±	12.0	0.590	0.559	0.18
Peak Braking Velocity (m/s)	12.9	±	15.8	16.3	±	12.5	0.767	0.447	0.24
Average Propulsion Velocity (m/s)	5.6	±	3.6	7.5	±	5.4	1.306	0.199	0.41
Peak Propulsion Velocity (m/s)	4.7	±	3.3	5.2	±	2.5	0.515	0.610	0.161

Data are Means ± SD.

**Table 4:** Percent changes from pre to post warmup by sex for average and peak braking and propulsion power-based metrics.

Power-Based Metric	Female % Δ			Males % Δ			*t*-Statistic	*p*-Value	Effect Size Cohen’s *d*
Average Braking Power (W)	17.7	±	18.7	25.1	±	17.9	1.281	0.208	0.40
Peak Braking Power (W)	16.9	±	20.0	26.1	±	18.1	1.540	0.132	0.48
Average Propulsive Power (W)	9.0	±	5.8	11.1	±	6.8	1.046	0.302	0.33
Peak Propulsive Power (W)	7.5	±	4.4	7.9	±	4.4	0.317	0.753	0.10

Data are Means ± SD.

**Table 5:** Illustrates percent changes from pre to post warmup by sex for grip strength, and various key performance indicators of CMJ performance.

Grip and CMJ Metrics	Female % Δ			Males % Δ			*t*-Statistic	*p*-Value	Effect Size Cohen’s *d*
Grip Strength (kg)	3.0	±	8.9	0.4	±	7.5	−1.039	0.305	−0.32
Jump Height (cm)	10.9	±	7.9	11.6	±	6.1	0.299	0.767	0.09
Flight Time (seconds)	5.0	±	3.1	5.7	±	3.2	0.728	0.471	0.23
Braking RFD (N/s)	26.0	±	38.0	44.6	±	47.3	1.379	0.176	0.43
Jump Momentum (Kg*m/s)	5.1	±	3.6	5.5	±	2.8	0.416	0.680	0.13
CMJ Depth (cm)	1.2	±	18.7	4.4	±	11.9	0.659	0.514	0.21

Data are Means ± SD.

**Table 6:** Regression statistics for force at minimum displacement, average braking force and peak braking force.

Dependent Variable	Model	*R* ^2^	Δ*R*^2^	Adj. *R*^2^	*F*	ANOVA *p*	β (Sex)	*t*	95% CI	
Force at Min Displacement	Sex	0.1	0.1	0.077	4.4	0.043	−0.317	−2.09	−11.390	−0.178
Force at Min Displacement	Sex + LBM	0.152	0.052	0.108	3.4	0.043	0.051	0.18	−9.540	11.401
Average Braking Force	Sex	0.143	0.143	0.121	6.5	0.015	−0.378	−2.55	−10.106	−1.159
Average Braking Force	Sex + LBM	0.165	0.022	0.121	3.8	0.032	−0.136	−0.48	−10.518	6.471
Peak Braking Force	Sex	0.101	0.101	0.077	4.4	0.043	−0.317	−2.09	−11.465	−0.182
Peak Braking Force	Sex + LBM	0.15	0.05	0.106	3.4	0.045	0.043	0.15	−9.763	11.338

## Data Availability

Due to university policy, the data supporting this study are not publicly available. Data access requests may be directed to the corresponding author, G.S., and will be evaluated in accordance with institutional policies and applicable regulatory requirements.
